# Perinatal Arterial Ischemic Stroke Related to Internal Carotid Artery Occlusion: Two Case Reports

**DOI:** 10.1002/ccr3.73060

**Published:** 2026-07-02

**Authors:** Jin Wang, Yanping Huang, Lingkong Zeng, Xuwei Tao, Shi Wang

**Affiliations:** ^1^ Department of Neonatology, Wuhan Children's Hospital (Wuhan Maternal and Child Healthcare Hospital), Tongji Medical College Huazhong University of Science & Technology Wuhan China

**Keywords:** case report, internal carotid artery occlusion, new, perinatal arterial ischemic stroke

## Abstract

Perinatal arterial ischemic stroke (PAIS) constitutes one of the leading causes of neurological deficits in children. Internal carotid artery occlusion (ICAO) represents a rare etiology of PAIS and has been documented in only a limited number of case reports. Herein, we present two cases of PAIS related to ICAO. Both neonates were discharged following resolution of acute clinical symptoms; however, follow‐up assessments revealed persistent contralateral limb hypokinesia. ICAO is a rare entity in neonates, characterized by insidious and atypical manifestations in the neonatal period, and is associated with relatively poor neurological outcomes. For neonates diagnosed with PAIS, comprehensive cervical vascular imaging should be performed to enable early and definitive diagnosis of neonatal ICAO.

## Key Clinical Message

Internal carotid artery occlusion is a rare neonatal disorder that may present as convulsive seizures and lethargy. Due to its insidious and atypical neonatal manifestations, the prognosis is relatively unfavorable. Early cervical vascular imaging is indispensable for accurate diagnosis and appropriate clinical management.

## 1 Introduction

Perinatal arterial ischemic stroke (PAIS) is defined as ischemic brain injury resulting from infarction of one or more cerebral artery branches due to diverse etiology factors, leading to impaired perfusion in the corresponding cerebral regions [1]. The incidence of PAIS ranges from 1/2300 to 1/4000 live births, with disease onset occurring from 20 weeks to 28 days after birth [2]. PAIS is primarily diagnosed via cranial magnetic resonance imaging (MRI) and represents a major cause of long‐term neurological impairments, including limb motor dysfunction and cognitive deficits, in affected children [1, 2, 3].

The core clinical manifestations of PAIS include convulsive seizures, lethargy, hypotonia, and other neurological symptoms [1]. The pathogenesis of PAIS involves two principal mechanisms. The first is placenta‐derived embolism, in which thrombi originating from the placenta traverse the fetal circulation, pass through the patent foramen ovale, resulting in an infarction of one or more cerebral artery branches [1, 2, 3]. The second mechanism is in situ arterial thrombosis secondary to direct vascular injury during the perinatal period, which ultimately culminates in ischemic stroke [1, 2, 3].

Internal carotid artery occlusion (ICAO) is an uncommon cause of PAIS and has been reported in only a small cohort of neonate cases [4, 5, 6]. Owing to the absence of routine angiographic sequences in standard neonatal stroke neuroimaging protocols—particularly at the cervical level—ICAO is identified in fewer than 0.5% of neonates with PAIS [5]. In this case series, we describe two newborns diagnosed with PAIS secondary to ICAO. All clinical procedures and investigations performed in these cases were approved by the Institutional Review Board of Wuhan Children's Hospital (Registration No. 2021R045‐E02).

## 2 Case History/Examination

The infant A was an 18‐h boy, born via vaginal delivery at 37 weeks and 5 days, weighed 2860 g and had an Apgar score of 8, 9, and 9 at 1, 5, and 10 min, respectively. His mother had an uncomplicated pregnancy. The infant presented with poor postnatal responsiveness, decreased movement of the right extremities, and subsequent worsening clonic seizures, prompting admission to the neonatal intensive care unit (NICU).

Infant B was an 8‐h‐old girl who was born via caesarean section at 39 weeks and 3 days of gestation, with a birth weight of 3000 g and Apgar scores of 8, 8, and 9 at 1, 5, and 10 min, respectively. Her mother had an uneventful pregnancy. The infant's predominant postnatal manifestations were tachypnea, depressed responsiveness, and hypotonia of the right upper and lower extremities.

## 3 Differential Diagnosis, Investigations and Treatment

### 3.1 Infant A

On the second postnatal day, cranial computed tomography (CT) demonstrated large, well‐demarcated hypodense lesions in the left temporoparieto‐occipital lobe, consistent with left frontotemporoparietal cerebral infarction. On the third postnatal day, cranial MRI revealed extensive left hemispheric ischemic stroke on T1‐weighted, T2‐weighted, and diffusion‐weighted imaging (DWI) sequences; magnetic resonance angiography (MRA) showed no overt abnormalities of the anterior cerebral arteries (ACA), middle cerebral arteries (MCA), posterior cerebral arteries (PCA), or their major branches; as shown in Figure 1a–d. Neck MRI and MRA revealed occlusion of the left internal carotid artery (ICA), and the right ICA and branches were not significantly abnormal, as shown in Figure 1e,f. Amplitude integrated video electroencephalogram (aEEG) showed moderate abnormalities consistent with gestational age, characterized by frequent multifocal sharp waves, sharp‐and‐slow wave complexes, spikes, and occasional monorhythmic discharges, predominantly over the bilateral central, midline central, and left occipital regions, with marked disruption of sleep architecture. Five clinical seizures and two electrographic seizures were detected. Laboratory evaluation revealed reduced protein C activity (33.00%, reference range: 60%–140%) and decreased protein S activity (48.91%, reference range: 63.5%–149%), remaining investigations (PLT, comprehensive thrombin test kit, TT, PT, APTT, and stroke panel) were unremarkable.

**FIGURE 1:**
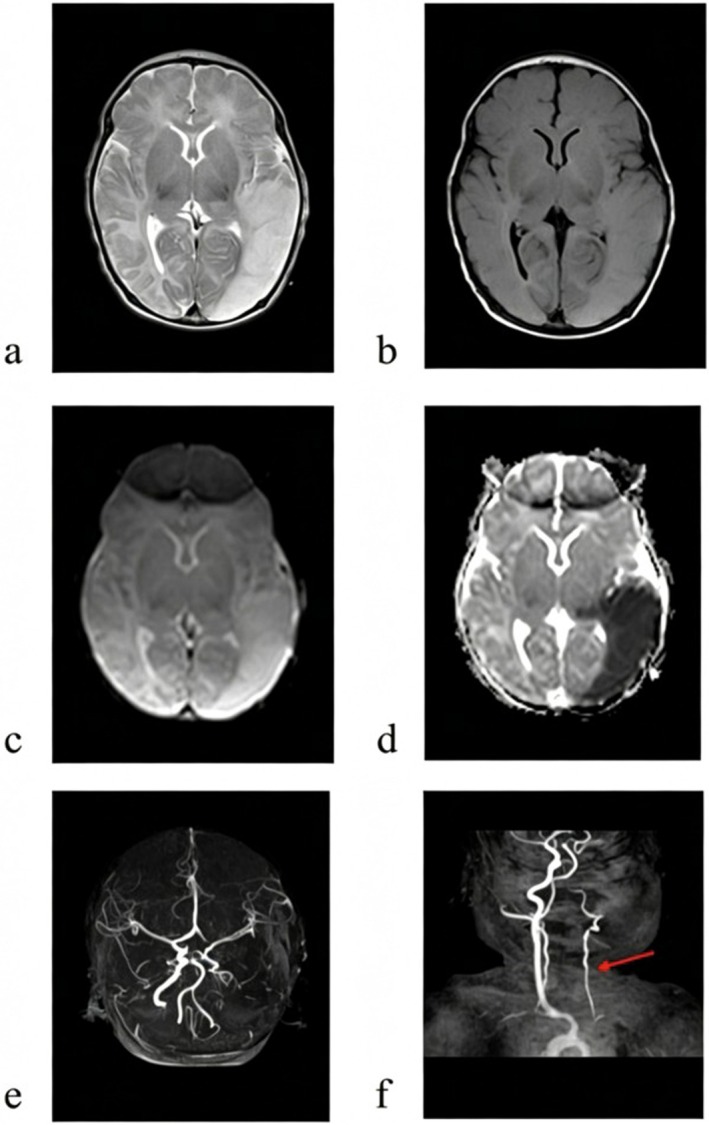
The result of MRI of infant A. Three‐day‐old male infant. (a–d) Brain MRI shows extensive left hemispheric ischemic infarction on T1WI, T2WI and DWI, intracranial MRA demonstrates normal ACA, MCA, PCA; (e, f) Neck MRA revealed occlusion of the left ICA (red arrows), with preserved right ICA.

The infant received no antithrombotic therapy during hospitalization. Supportive care included ceftazidime for antimicrobial prophylaxis and phenobarbital for seizure control, with subsequent resolution of clonic seizures and improved responsiveness. Follow‐up aEEG showed no obvious electrographic seizures. Oral phenobarbital was continued at discharge. At 6‐month follow‐up, no recurrent seizures were observed; the infant achieved independent sitting and rolling over. However, he exhibited persistent marked hypokinesia of the right upper and lower extremities compared with the left side.

### 3.2 Infant B

Immediate postnatal cranial CT demonstrated swelling of the left frontal parietal lobe, basal ganglia, and thalamus, consistent with cerebral infarction. On the first postnatal day, cranial MRI confirmed extensive left hemispheric ischemic stroke on T1‐weighted, T2‐weighted, and DWI sequences. On the second postnatal day, cranial MRA revealed occlusion of the left ICA and left MCA, with no abnormalities of other cerebral artery branches. Magnetic resonance venography (MRV) was unremarkable, as shown in Figure 2. Coagulation profile was normal; laboratory testing showed reduced protein C activity (26.30%, reference range: 60%–140%) and decreased protein S activity (36.55%, reference range: 63.5%–149%).

**FIGURE 2:**
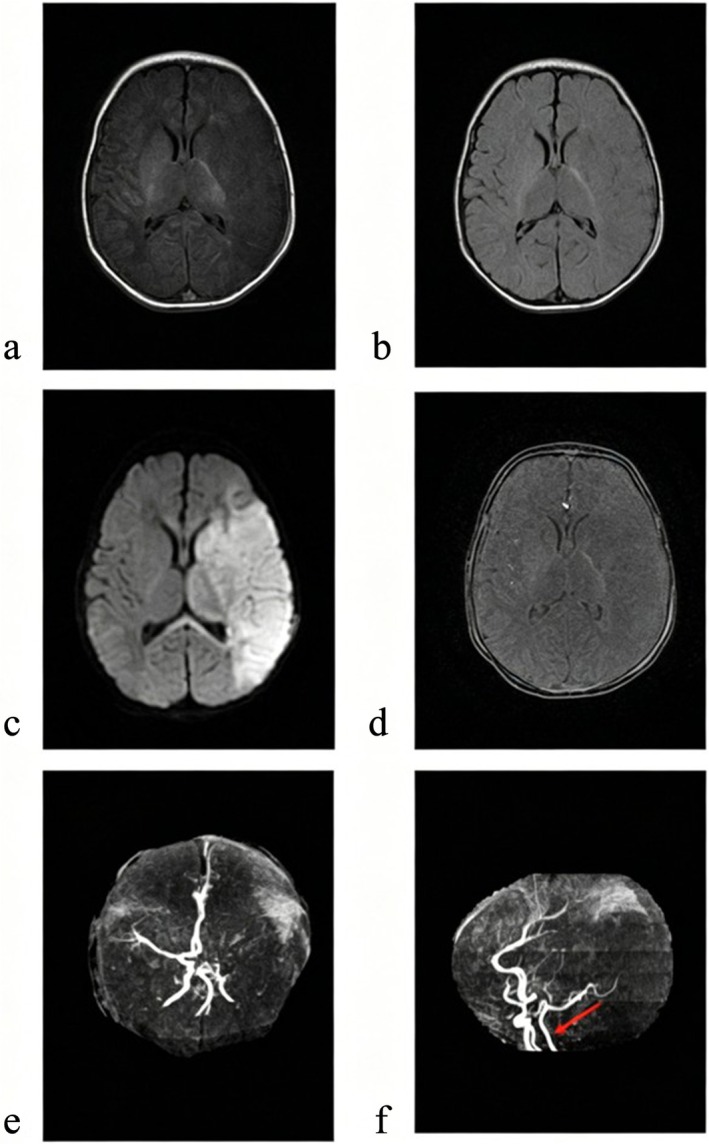
The result of MRI of infant B. One‐day‐old female infant. (a–d) Extensive left hemispheric ischemic infarction on T1WI, T2WI, and DWI. (e, f) MRA revealed occlusion of the left ICA and left MCA (red arrows); other cerebral arteries are unremarkable.

The infant B received neither anticonvulsant nor antithrombotic therapy during hospitalization. Supportive management included ampicillin and ceftazidime for antimicrobial prophylaxis, fluid resuscitation, and noninvasive respiratory support. The infant was discharged following clinical improvement. At 6‐month follow‐up; however, no seizures were documented; however, persistent hypotonia and marked hypokinesia of the right upper and lower extremities relative to the contralateral side were noted.

## 4 Conclusion and Results (Outcome and Follow‐Up)

Both infants exhibited gradual clinical improvement with standardized supportive care. Despite confirmed ICAO, neither neonate received thrombolytic or anticoagulant therapy. Both received short‐term anticonvulsant therapy during hospitalization, which was discontinued following seizure control. Longitudinal follow‐up demonstrated residual contralateral limb motor dysfunction in both neonates.

## 5 Discussion

ICAO is a major etiological factor underlying cerebral ischemic events including arterial ischemic stroke. This condition predominantly affects adults, with rare reports in children and neonates; large‐scale epidemiological data on its incidence in the neonatal population remain lacking. Based on several large clinical analyses of PAIS, the current incidence in newborns is less than 0.5% [5]. The clinical presentation of ICAO is heterogeneous, and affected individuals may be asymptomatic [5]. Symptomatic patients typically present with hemiplegia, transient ischemic attack (TIA), or acute ischemic stroke [1, 2, 3].

Based on our case series and literature review, the predominant neonatal manifestations of ICAO include focal seizures and depressed responsiveness. However, the high rates of disability and mortality, coupled with atypical neonatal clinical features, contribute to a high risk of missed diagnosis [5]. Commonly utilized imaging modalities for diagnosing ICAO include ultrasonography, computed tomography angiography (CTA), MRA, digital subtraction angiography (DSA), and high‐resolution MRI (HR‐MRI) [5]. Ultrasonography serves as a useful screening tool but has limited sensitivity for detecting intracranial and distal ICA occlusion [5]. Although DSA remains the gold standard for diagnosing ICAO, CTA and MRA are the most widely employed modalities in clinical practice due to the high radiation exposure associated with DSA. MRA is particularly favored given its noninvasive nature and relatively low radiation burden [4, 5, 6].

ICAO‐related cerebral infarction can be categorized into intracortical infarction, subcortical infarction, watershed infarction and large hemispheric infarction based noncranial CT or MRI findings [7, 8]. Unilateral ICA thromboembolism may occlude the ipsilateral ACA and MCA, or extend to the contralateral ACA and MCA via the circle of Willis, resulting in regional infarction and subsequent clinical manifestations including aphasia, hemiplegia and sensory deficits [9, 10]. In this series, both neonates presented with left ICAO, leading to ipsilateral cerebral hemisphere infarction and subsequent seizure activity.

A comprehensive literature review [5, 6, 7, 8, 9, 10, 11, 12, 13, 14, 15] identified only one reported case of bilateral ICAO, among 17 neonates with unilateral ICAO; all developed ipsilateral cerebral artery ischemic stroke, with the MCA territory most commonly involved [5]. Although the MCA is frequently occluded, collateral perfusion via the anterior and posterior communicating arteries (A‐com and P‐com) may maintain blood flow to the ipsilateral MCA territory. In infant A, this collateral circulation likely resulted in a relatively normal appearance on initial cranial MRA. In contrast, infant B exhibited hypoplasia of the left A1 segment of the ACA and the left P‐com, which failed to provide adequate collateral supply to the left MCA territory, leading to concurrent occlusion of the left ICA and MCA on MRA.

ICAO is classified into four subtypes based on angiographic features: Type A: conical proximal ICA occlusion with a residual lumen; Type B: non‐conical proximal ICA occlusion with a residual lumen; Type C: complete proximal ICA occlusion, with the common carotid artery directly into the external carotid artery and vascular remodeling of the cavernous or petrosal segment; Type D: complete proximal ICA occlusion, with the common carotid artery continuing directly into the external carotid artery and no vascular remodeling of the cavernous or petrous segments [16, 17, 18, 19]. In adults, type A and B occlusion are associated with higher recanalization success rates and lower complication rates, making them more amenable to surgical intervention [19]. In published neonatal cases, ICAO subtype could not be definitively classified due to incomplete documentation of ICA flow dynamics. The imaging findings of the infants in our cases were consistent with type A ICAO.

The management of ICAO includes conservative medical therapy and surgical intervention. Medical therapy primarily consists of antiplatelet agents with or without statin therapy. In the adult population, combination antiplatelet and statin therapy is superior to antiplatelet monotherapy for reducing the risk of silent embolism and recurrent stroke [17, 18]. However, these studies focus predominantly on adult patients with underlying comorbidities such as hyperlipidemia. As most children lack such risk factors, high‐quality clinical data evaluating combination medical therapy for pediatric and neonatal ICAO are unavailable.

Surgical interventions include carotid endarterectomy (CEA), extraction‐intracranial bypass (EI‐CI bypass), percutaneous artery stenting (CAS), endovascular intervention (EI), angioplasty, composite procedures [12, 13]. However, surgical therapies are primarily utilized in adults and carry risks of vascular injury, cerebral parenchymal hemorrhage, and subarachnoid hemorrhage, as well as a substantial risk of recurrent stroke and high mortality; thus, surgical intervention is rarely performed in children [17, 18]. In our series, the uncertain timing of ICAO and stable occlusion on serial vascular ultrasound and other imaging studies, together with evidence of infarct expansion on serial cranial imaging, precluded the need for interventional or surgical therapy. Both infants improved clinically with supportive care including antiepileptic therapy and fluid management. Published pediatric case reports describe supportive care including antiepileptic, anticoagulant, and nutritional support; no report of thrombolytic or surgical therapy in neonates was identified, consistent with our findings.

Etiologies of ICAO include traumatic, atherosclerotic, iatrogenic, and idiopathic causes. In adults, atherosclerosis is the predominant cause; in children, ICAO is most frequently attributed to iatrogenic, traumatic, or idiopathic factors. Iatrogenic causes are primarily related to giant ICA aneurysm shunting procedures, while traumatic causes include falls and pharyngeal trauma [20, 21, 22, 23]. The etiology of neonatal ICAO remains incompletely understood; proposed risk factors include: ① Direct ICA injury secondary to macrosomia and shoulder dystocia [5, 13], although large‐scale data confirming this association are lacking. ② Placental pathology: placenta plays an important role in the development of ICAO [5]. More than 80% of neonates with PAIS exhibit placental thrombosis and placental inflammation [24]. ③ Perinatal coagulation abnormalities: neonates demonstrate physiologically low levels of anticoagulant protein C and protein S, which may confer a prothrombotic state [25].

In our cases, both mothers had uncomplicated pregnancies and deliveries, with normal Apgar scores. Placental pathological examination was not performed postpartum; thus, the presence of placental thrombosis or inflammation cannot be confirmed. Laboratory evaluation on admission revealed reduced protein C and protein S activity, which normalized on post‐discharge follow up. These findings suggest that perinatal coagulation abnormalities may represent the primary etiology of ICAO in these neonates.

Fetal macrosomia, shoulder dystocia, placental abnormalities, and neonatal coagulopathy may therefore contribute to the development of neonatal ICAO, presenting new challenges for neurological monitoring in the NICU. In conclusion, ICAO is a rare neonatal disorder characterized by insidious and atypical manifestations and relatively poor neurological outcomes. For neonates diagnosed with PAIS, comprehensive cervical vascular imaging is strongly recommended to enable early and accurate diagnosis of ICAO.

## Author Contributions

**Jin Wang:** conceptualization, writing – original draft, writing – review and editing. **Yanping Huang:** conceptualization, writing – original draft, writing – review and editing. **Lingkong Zeng:** conceptualization, project administration. **Xuwei Tao:** methodology, visualization, writing – original draft. **Shi Wang:** methodology, validation, visualization.

## Funding

This work was supported by the Natural Science Foundation of Hubei Province (2023AF888) and the Funding for Scientific Research Projects from Wuhan Municipal Health Commission (WX 23A91).

## Ethics Statement

This study was reviewed and approved by the Ethics Committee of Wuhan Children's Hospital (Wuhan Maternal and Child Healthcare Hospital), Tongji Medical College, Huazhong University of Science & Technology (registration No. 2021R045‐E02). The guardians of the children provided written informed consent for the clinical study.

## Consent

Written informed consent was obtained from the patients for publication of the details of their medical case and any accompanying images.

## Conflicts of Interest

The authors declare no conflicts of interest.

## Data Availability

The original contributions presented in this study are included in the article/Supporting Information, and further inquiries can be directed to the corresponding author.
